# Supplementary material for: Frequency and diversity of carbapenemase-producing Enterobacterales recovered from untreated wastewater impacted by selective media containing cefotaxime and meropenem in Ohio, USA
Source: PLoS One. 2023 Feb 22;18(2):e0281909. doi: 10.1371/journal.pone.0281909 (PMC9946209; doi:10.1371/journal.pone.0281909)
Supplement: Attachment — Submitted filename: Response to the Reviewers. 24-12-2022. docx.docx [file pone.0281909.s001.docx]

**6^th^ December 2022**

**Re: Response to Reviewers Updated.**

I hereby appreciate all the PLOS ONE Secretariat and the reviewers; one (1) and two (2) for the good review comments raised and suggestions to improve this manuscript. They were very helpful. Thank you.

The comments raised were addressed as follows;

**Note**: Corresponding email; changed to **too.4@osu.edu**

1. **Plos -One style requirements-** All the files are named accordingly.
2. **PLOS questionnaire on Inclusivity –** included as an attachment.
3. **Financial disclosure;**
a. I am a Ph.D. student at the University of Nairobi, OHEART – GOHi Ph.D. fellow at the Ohio State University (OSU) through the National Institutes of Health (NIH) Fogarty international center (grant number TW008650) This was a mentored Training through Ph.D. fellowship exchange program involving the three institutes (OSU, KEMRI and the University of Nairobi).
b. The funders had no role in study design, data collection, and analysis, decision to publish, or preparation of the manuscript
c. 1^st^ Author student stipend (National Institutes of Health (NIH) Fogarty international center (grant number TW008650)
d. The authors received no specific funding for this work
4. **Financial information:**
   a. Rael Too (1^st^ and corresponding author)
   b. grant number TW008650
   c. Student stipend
5. **Data Availability statement;**

- The original contribution presented in this study is included in the study manuscript, further inquiries can be directed to the corresponding author.

6. **Figure 1&2. No figures, (All deleted)**
7. There is **no figure 2**, and any references to it are **deleted and revised**
8. **Tables -**The Tables are corrected and captioned correctly see (**Tables 1, 2, 3, 4, 5)**
9. **Reference list**

The reference list (**font changed -Times Roman 12**), **duplicate references edited** additional references added and the list updated;

1. Azuma T, Otomo K, Kunitou M, Shimizu M, Hosomaru K, Mikata S, et al. Environmental fate of pharmaceutical compounds and antimicrobial-resistant bacteria in hospital effluents, and contributions to pollutant loads in the surface waters in Japan. Sci Total Environ. 2019 Mar;657:476–84.
2. Proia L, Anzil A, Borrego C, Farrè M, Llorca M, Sanchis J, et al. Occurrence and persistence of carbapenemases genes in hospital and wastewater treatment plants and propagation in the receiving river. J Hazard Mater. 2018 Sep;358:33–43.
3. Cahill N, O’Connor L, Mahon B, Varley Á, McGrath E, Ryan P, et al. Hospital effluent: A reservoir for carbapenemase-producing Enterobacterales? Sci Total Environ. 2019 Jul;672:618–24.
4. Suzuki Y, Nazareno PJ, Nakano R, Mondoy M, Nakano A, Bugayong MP, et al. Environmental Presence and Genetic Characteristics of Carbapenemase-Producing *Enterobacteriaceae* from Hospital Sewage and River Water in the Philippines. Elkins CA, editor. Appl Environ Microbiol. 2020 Jan 7;86(2):e01906-19.
5. Pauwels B, Verstraete W. The treatment of hospital wastewater: an appraisal. J Water Health. 2006 Dec 1;4(4):405–16
6. Cornaglia G, Akova M, Amicosante G, Cantón R, Cauda R, Docquier JD, et al. Metallo-β-lactamases as emerging resistance determinants in Gram-negative pathogens: open issues. Int J Antimicrob Agents. 2007 Apr;29(4):380–8.

**Additional Editor comments**

1. **Tables**

There were only five tables presented in the manuscript and all are renumbered and now correspond to the information in the text. “Table 1” in the text preceding included (**page 4 line-150)**

2. Discussion section - **rewritten**
3. Limitations of this present study included (page **9 lines 281-296)**
4. **Review comments to the author**
A. **Reviewer #1: the full objective of the study,**
i. “Testing the concentration of the drugs in addition to different types of drugs”

**Only two β-lactam antibiotics were being tested (meropenem and cefotaxime)**

ii. ‘How did Meropenem concentrations fair but fail to provide different (**Line 243-249)** concentrations of cefotaxime, were they testing meropenem concentration?’ **Yes, we tested Meropenem concentration at 0.5 μg/mL and 1.0 μg/mL meropenem modified with 70µg/mL ZnSO_4_ to check what concentration was ideal to recover more CPEs.**
iii. Line 228 specific concentrations of Meropenem, and cefotaxime showed good results without providing data on cefotaxime. **Cefotaxime had low recovery rate compared to Meropenem in MacConkey broth** (**Line 243-249)**
iv. Details on DNA extraction method included **(page 4, lines 139-144)**
B. Reviewer #2: AMR threats
i. Background data on AMR consumption in Hospitals/areas contributing to the wastewater tested in the study. (**Done- Introduction section lines 61-65)**
ii. Data that shows the area studied has high antibiotic consumption will be helpful. (**Included lines 216-222)**
iii. Table labeling in the manuscript **Addressed**

**NB: Additional comments raised in recent submissions R1 and edits from the	Supervisor**

About the mismatch in financial disclosure and financial information, this has been	addressed as follows;

**Financial disclosure;**

a) I am a Ph.D. student at the University of Nairobi, OHEART – GOHi NIH D43 Ph.D. fellow at the Ohio State University (OSU) through the National Institutes of Health (NIH) Fogarty international center (grant number TW008650) This was a mentored Training through Ph.D. fellowship exchange program involving the three institutes (OSU, KEMRI and the University of Nairobi).
b) The funders had no role in study design, data collection, and analysis, decision to publish, or preparation of the manuscript
c) 1^st^ Author student stipend (National Institutes of Health (NIH) Fogarty international center (grant number TW008650)
d) The authors received no specific funding for this work

**Financial information:**

a. Rael Too (1^st^ and corresponding author)
b. grant number TW008650 (NIH D43 Fogarty Fellow GOHi-OHEART-OSU)
c. Student stipend

**Other comments raised by the last author and have been addressed includes;**

a. Title edited to read; **“*Frequency and diversity of Carbapenemase-producing Enterobacterales recovered from untreated wastewater impacted by selective media containing cefotaxime and meropenem in Ohio, USA***.”
b. **replaced** *Enterobacteriaceae***- *Enterobacterales*** through out the manuscript**.**
c. Minor typos in throughout the Manuscript (**Final Markedup copy with track changes)**
