# Supplementary material for: Frequency and diversity of carbapenemase-producing Enterobacterales recovered from untreated wastewater impacted by selective media containing cefotaxime and meropenem in Ohio, USA
Source: PLoS One. 2023 Feb 22;18(2):e0281909. doi: 10.1371/journal.pone.0281909 (PMC9946209; doi:10.1371/journal.pone.0281909)
Supplement: Attachment — Submitted filename: Response to the Reviewers -14th January 2023.docx [file pone.0281909.s002.docx]

**14^th^ January 2023**

**Response to Reviewers Manuscript R2.**

Greetings! I hereby appreciate all of you the PLOS ONE Secretariat and the reviewers; one (1) and two (2) for the good review comments raised to improve this paper. They were very helpful. Thank you.

1. **Laboratory protocols- N/A**
2. **Discussion -** Done
   i. **Repeated values deleted and others used percentages to discuss.**
   ii. **Mentioned tables deleted**
   iii. **The discussion section is revised**
3. **Reference list**

The reference list is complete and correct

**Additional Editor comments**

1. **Review comments**
i. **Reviewer #2:**
i. There was no background data collected on AMR consumption in hospitals/areas contributing to the wastewater tested in the study.
ii. This is one of the limitations where by background data is missing or would have been collected and compare the results to ascertain the source **(Line 286-290).** However, this is a research gap and is part of the ongoing study in Kenya.
iii. Discussion section revised.

**N/B**: The findings from this study informed on the ideal method to use in implementing the current study in Kenya.

Looking forward to publication of this paper

Much Appreciated your continued support!
